# Mental health status among prison officers in the process of enforcing the law during COVID-19epidemic: a cross-sectional survey from China

**DOI:** 10.1186/s12888-021-03679-0

**Published:** 2022-01-11

**Authors:** Yang Li, Zhen Wen, Yimei He, Jingting Huang

**Affiliations:** 1 School of Law, Minzu University of China, Beijing, 100081 People’s Republic of China; 2 Department of General Surgery, Chengdu Third People’s Hospital, Chengdu, 610031 People’s Republic of China; 3 Dong Cheng Experimental Junior Middle School, Guangyuan, 628017 People’s Republic of China; 4 West China School of Medicine, Sichuan University, Chengdu, 610041 People’s Republic of China

**Keywords:** COVID-19, Execution of punishment, Mental health, Frontline prison officers, Administrative governance

## Abstract

**Background:**

A global public health emergency triggered by the Coronavirus Disease 2019 (COVID-19) epidemic may have are markable psychological impact on the population. There is still limited psychological research on police officers, especially prison officers in the process of enforcing the law. The present study aims to identify prevalence and influencing factors on mental health status among frontline prison officers in China during the prevention and control of the COVID-19 epidemic.

**Methods:**

A cross-sectional survey with a sample of 981 frontline prison officers was conducted using snowball sampling approach. The self-administered questionnaire consisted of 4 parts: (i) informed consent form; (ii) socio-demographic section; (iii) work and life situations during the prevention and control of the COVID-19 epidemic; (iv) the Chinese version of the 12-item General Health Questionnaire (GHQ-12). Univariate analysis and multivariable logistic regression were performed to identify factors influencing mental health status.

**Results:**

The prevalence of being prone to mental health problems (GHQ-12 score ≥ 4) was 33.43% among frontline prison officers. The results of GHQ-12 factors analysis indicated that the prison officers suffered from psychological issues was related to anxiety and depression, which main symptoms were unhappy and depressed, lost sleep over worry and constantly under strain. Multivariate logistic regression analysis revealed that male (OR = 1.573, 95% CI:1.385–1.853), lockdown shift inside the prison(OR = 2.203, 95% CI:2.139–2.297), more night shifts (OR = 2.163, 95% CI:2.031–2.317; OR = 2.749, 95% CI:2.194–2.901), more smoking (OR = 1.100, 95% CI:1.037–2.168), poor self-reported physical condition (OR = 1.947, 95% CI:1.478–2.250), chronic or serious illness history(OR = 1.870, 95% CI:1.314–2.660; OR = 2.214, 95% CI:1.460–2.812) were risk factors for mental health among frontline prison officers, while regular diet (OR = 0.779, 95% CI:0.539–0.928), more physical exercise (OR = 0.702, 95% CI:0.548–0.899; OR = 0.641, 95% CI:0.316–0.887), more communication with family members (OR = 0.437, 95% CI:0.295–0.616) were protective factors.

**Conclusion:**

Chinese frontline prison officers experienced different psychological stress coming from the prevention and control of this epidemic. Therefore, continued surveillance of psychological problems and targeted mental health care for frontline prison officers were urgent.

**Supplementary Information:**

The online version contains supplementary material available at 10.1186/s12888-021-03679-0.

## Background

On January 30, 2020, the epidemic was declared a global public health emergency by the WHO Emergency Committee [1]. There have been more than 100 million confirmed cases, including 2,147,411 deaths worldwide as of January26, 2021 [2]. Countries took different safety measures and precautions to limit the spread and risk of the epidemic, such as obligatory lockdowns, travel was halted, airports were shutdown, many work spaces, schools and universities were closed, and prison visits were cancelled [3, 4]. The crisis of public health crisis triggered by exposing to the COVID-19 epidemic and the preventive taken to control infections have become the major threats to both physical and mental health of the population, especially to frontline workers [5–7].

In the Chinese prison system, each prison has its own prison hospital to provide health services, and prison health services are led by the Ministry of Justice and guided by the Ministry of Health. Local prisons in China are directly managed by the Provincial Prison Bureau, and supervised by supervisory organs, people’s congresses and the general public in the process of strictly implementing the Criminal Law, Criminal Procedure Law and Prison Law of the People’s Republic of China. Chinese prisons only hold convicted prisoners, which have been sentenced to fixed-term imprisonment, life imprisonment or death with a two-year reprieve [8]. It’s worth mentioning that overpopulation detention is not an issue in China; thus, the prisons are able to avoid many consequences such as the increased risk of spread and the need for additional prisoner release [9].

As a result of the rapid spread of the epidemic, prisons in several countries have experienced a serious problem with COVID-19infection. For example, confirmed cases were reported in prisons of British, France, United States, Pakistani, India and Brazil, etc. [10–14]. Lessons from COVID-19 infections in both domestic and foreign prisons were learned deeply by the penitentiary system in China. The prison authorities in western China have formulated and implemented many effective measures to prevent and control the epidemic, and achieved major results of no infections, no outbreaks, and no deaths [15]. This achievement was obtained mainly from the perseverance and dedication of frontline prison officers. The work patterns of Chinese prison officers have been divided into three types during the prevention and control of the COVID-19 epidemic, which were lockdown shift inside the prison, quarantine at designated location, and a lockdown resting period at home [15]. The Chinese prison officers were ordered to fight at the forefront of pandemic control in prisons by working on shifts inside for an extended and indefinite period of time, which proved effective in terminating the spread of the virus. The strategy of Chinese prison system provides useful exploration for the epidemic prevention work of prisons in the world. However, the stressful task of preventing the COVID-19 epidemic and supervising criminals placed a heavy burden on the personal lives of the officers [9].

During pandemic outbreaks, frontline workers including healthcare workers and police staff were under unprecedented pressure from the heavy workload, risky workplace environment, and non-availability of adequate leaves/duty off periods [5, 7, 16]. These severe situations caused them to be especially vulnerable to mental health issues, such as anxiety and depression symptoms [17, 18].The psychological problems would negatively interfere with the efficiency of frontline workers to fight the epidemic, and may pose a threat to their overall well-being. Previous studies have acknowledged concerns about the psychological problems of frontline doctors, nurses and paramedical staffs [5, 6, 18], the psychological assistance for healthcare workers have been provided by many countries, and the implementation facilitators to supporting programmes for improving the resilience and mental health of frontline workers during and after COVID-19 epidemic [18]. A latest study also showed that COVID-19 duties during the lockdown period of police personnel exhibited significant anxiety, depression symptoms, and perceived significant stress [16]. However, there is still no systematic research involving the mental health of prison officers.

Across-sectional survey has been conducted to investigate the psychological issues among frontline prison officers during the prevention and control of the COVID-19 epidemic, and multivariable logistic regression is used to determine the factors influencing mental health status. The aim of the current study was also to explore which factors may influence the mental health of prison officers, which might have a certain impact on their enforcement of relevant laws. These findings will aid local governments and prison administrators to formulate effective policies, regulations and actionable psychological interventions for promoting mental wellbeing of frontline prison officers in public health emergencies.

## Methods

### Study design and participants

A cross-sectional study was implemented using anonymous electronic questionnaires through an online survey platform called “Questionnaire Star” from March 28 to April 20, 2020. These questionnaires were distributed to participants via WeChat, which is the most widely used social networking software in China, using a snowball sampling approach. We encouraged participants to send the questionnaire link to their colleagues whom they considered suitable for this survey. The purpose of the survey lasting for nearly a month was to investigate as much as possible subjects who have experienced all work patterns during the prevention and control of the COVID-19 epidemic. The participants of present study comprised of western Chinese frontline prison officers in the process of enforcing the law. Those who have been diagnosed with a history of mental disorder, refused to participate in survey, and unable to operates mart phone or computer devices were excluded.

### Sample size and sampling procedure

Following formula was used to calculate the appropriate sample size for this study [19]. The minimum sample size (*n*) was estimated according to the following parameters: N was the relative number of prison officers in western China, design effect (DEFF) was set at 2.0,Z_1 − α/2_was set at 2.58 (level of significance 99% with the two-tailed test), p was set at 4% based on the prevalence of psychological problems (3.6–5.0%) with Chinese adults in an earlier survey [20], and the sample error(d) was 5%.

$$n=\left[ DEFF\ast N\ast p\ast \left(1-p\right)\right]/\left[{d}^2/{{\mathrm{Z}}^2}_{1-\upalpha /2}\ast \left(N-1\right)+p\ast \left(1-p\right)\right]$$

The recommended sample size was calculated as 814 respondents. To account for the anticipated non-response rate, the questionnaire was distributed to 1035 prison officers. Finally, a total of 981 valid questionnaires as a convenience sample were completed without assistance.

### Data collection and survey questionnaire

Data were collected using self-administered questionnaire, which evaluated the mental health status of frontline prison officers in the phase of the COVID-19 epidemic. The survey questionnaire was developed with 4 parts: informed consent, socio-demographic factors, items on work and life situation of frontline prison officers, and mental health status. (i) Participants were informed that the right to take a voluntary participation was absolute, and the completion of the questionnaire implied providing the consent. (ii) Demographic characteristics of the participants included variables such as gender, age, marital status, education, and working years, etc. (iii)A self-designed questionnaire was used to collected the information about recent work patterns (quarantine at designated location, lockdown shift inside the prison, a lockdown resting period at home),night shifts (less than 7, 7 to 15, more than 15times/month), diet (irregular or regular), physical exercise (no exercise, less than 3, more than or equal to 3 days/week), smoking (never, sometimes, everyday), self-reported physical condition (good or poor), disease history in the past year (no illness, chronic, serious illness. It did not include mental health problems), and communication with family members (0 to 2, more than 2 times/week). (iv) The Chinese version of the 12-item General Health Questionnaire (GHQ-12) was used to measure mental health status in present study.

TheGHQ-12could be analyzed as a single dimension mental health test [21]. However, many factor-analytic studies have suggested that it could be divided into two or three specific and meaningful factors, in which each factor was composed of several items from the scale [22–24]. When compared to other methods and applied to different samples, the three-factor model proposed by Gribbin and Worsley(1977) [22], including anxiety and depression (Factor I, item of 2, 5, 6, and 9), social dysfunction (Factor II, item of 1, 3, 4, 7, 8, and 12), and loss of confidence (Factor III, item of 10 and 11), was verified to give the best fit [25]. Therefore, this model was used to measure the types of psychological issues that the frontline prison officers might suffer from. The GHQ-12 scale composed of 12 items with answers scored from “better than usual / not at all = 0”and“same as usual / no more than usual=0” to “less than usual / rather more than usual =1”and“much less than usual / much more than usual =1” [26]. All item scores were added to give GHQ-12 total score, which the possible score ranged from 0 to 12. The scale with higher total scores indicated higher degrees of disturbance of the mental health status [27]. In this study, the GHQ-12 was reliable and repeatable and showed good internal consistency (Cronbach’s alpha was 0.895). An additional table file shows this questionnaire in more detail (see Additional file 1).

### Ethical considerations

This study protocol was approved by the Ethical Review Board of West China Medical School at Sichuan University. All participants have signed the informed consent form via the “Questionnaire Star” after explaining the purpose of the study. Their personal information was collected anonymously, and all the data was used for research purposes only.

### Statistical analysis

Statistical analysis was performed with SPSS 24.0 software (SPSS Inc., Chicago, IL, USA).Enumeration data was presented as the numbers (*n*) and percentages (%). Measurement data of the GHQ-12 score were computed using the means (*SD*). Univariable logistic regression was performed to explore association of each categorical variable with the outcome using chi-squared (χ^2^) test. Multivariable logistic regression was used to determine the association of multiple potential predictors (independent variables) with mental health (dependent variable) by calculating the odds ratios(OR) and their 95% confidence interval (95% CI). The independent variables with *p*-value < 0.05 in univariable analysis were subsequently selected in the final multivariable model [28]. Backward likelihood ratio elimination method was used to make final effect model, and the results were expressed as standardized regression coefficient (*β*) and the odds ratios with 95% confidence intervals [OR (95% CI)]. Data was considered statistically significant at the level of a *p-*valueless than 0.05.

## Results

### General characteristics of the participants

A total of 981 frontline prison officers, including 262 (26.71%) of female and 719 (73.29%) of male, participated in this study with the response rate of 94.78%. The age of the respondents ranged from 20 to 57 years old, which of the mean age was (29.56 ± 6.13) years old. The marital status of being married (46.59%) was similar to that of being single (53.41%). Majority of the sample had a bachelor degree and above (73.80%), and 26.20% had a college degree and below.55.56% have been working less than 5 years in their profession, followed by between 5 years and 10 years (30.17%). Table 1 presented the general characteristics of the participants, and the groups significantly differed on gender, working years, recent work mode, night shifts,diet, physical exercise, smoking, self-reported physical condition, disease history in the past year, and communication with family members (all *p*-value< 0.05).

**Table 1:** Characteristics and mental health status of frontline prison officers [*n* (%)]

Variables	Grouping	Total sample	Mental health problems		*χ*^2^	*p*-value
			With (GHQ-12 score ≥ 4)	Without (GHQ-12 score < 4)		
Gender	Female	262 (26.71%)	70 (26.72%)	192 (73.28%)	6.435	*0.011
	Male	719 (73.29%)	254 (35.33%)	465 (64.67%)		
Age (years)	< 30	692 (70.54%)	225 (32.51%)	467 (67.49%)	2.202	0.333
	30–50	241 (24.57%)	91 (37.76%)	150 (62.24%)		
	> 50	48 (4.89%)	16 (33.33%)	32 (66.67%)		
	Mean ± *SD*	29.56 ± 6.13				
Marital status	Married	457 (46.59%)	151 (33.04%)	306 (66.96%)	0.060	0.807
	Single	524 (53.41%)	177 (33.78%)	347 (66.22%)		
Education	College degree and below	257 (26.20%)	96 (37.35%)	161 (62.65%)	2.403	0.121
	Bachelor degree and above	724 (73.80%)	232 (32.04%)	492 (67.96%)		
Working years	< 5	545 (55.56%)	233 (42.75%)	312 (57.25%)	8.460	*0.015
	5–10	296 (30.17%)	104 (35.14%)	192 (64.86%)		
	> 10	140 (14.27%)	44 (31.43%)	96 (68.57%)		
Recent working mode	A lockdown resting period at home	189 (19.27%)	20 (10.58%)	169 (89.42%)	167.576	** < 0.001
	Quarantine at designated location	379 (38.63%)	77 (20.32%)	302 (79.68%)		
	Lockdown shift inside the prison	413 (42.10%)	231 (55.93%)	182 (44.07%)		
Number of night shifts (times/month)	< 7	573 (58.41%)	138 (24.08%)	435 (75.92%)	58.459	** < 0.001
	7–15	296 (30.17%)	129 (43.58%)	167 (56.42%)		
	> 15	112 (11.42%)	61 (54.46%)	51 (45.54%)		
Diet	Irregular	249 (25.38%)	127 (51.00%)	122 (49.00%)	46.280	** < 0.001
	Regular	732 (74.62%)	201 (27.46%)	531 (72.54%)		
Physical exercise (days/week)	No exercise	633 (64.53%)	235 (37.12%)	398 (62.88%)	10.972	**0.004
	≤ 2	232 (23.65%)	61 (26.29%)	171 (73.71%)		
	≥ 3	116 (11.82%)	32 (27.59%)	84 (72.41%)		
Smoking	Never	499 (50.87%)	147 (29.46%)	352 (70.54%)	14.548	**0.001
	Sometimes	253 (25.79%)	81 (32.02%)	172 (67.98%)		
	Everyday	229 (23.34%)	100 (43.67%)	129 (56.33%)		
Self-reported physical condition	Good	846 (86.24%)	219 (25.89%)	627 (74.11%)	157.401	** < 0.001
	Poor	135 (13.76%)	109 (80.74%)	26 (19.26%)		
Disease history in the past year	No illness	830 (84.61%)	241 (29.04%)	589 (70.96%)	52.960	** < 0.001
	Chronic	133 (13.56%)	72 (54.14%)	61 (45.86%)		
	Serious illness	18 (1.83%)	15 (83.33%)	3 (16.67%)		
Communication with family members (times/week)	0–2	273 (27.83%)	125 (45.79%)	148 (54.21%)	25.932	** < 0.001
	≥ 3	708 (72.17%)	203 (28.67%)	505 (71.33%)		

**significant at *p* < 0.01, *significant at *p* < 0.05

### Prevalence of self-reported mental health problem

**Fig. 1:**
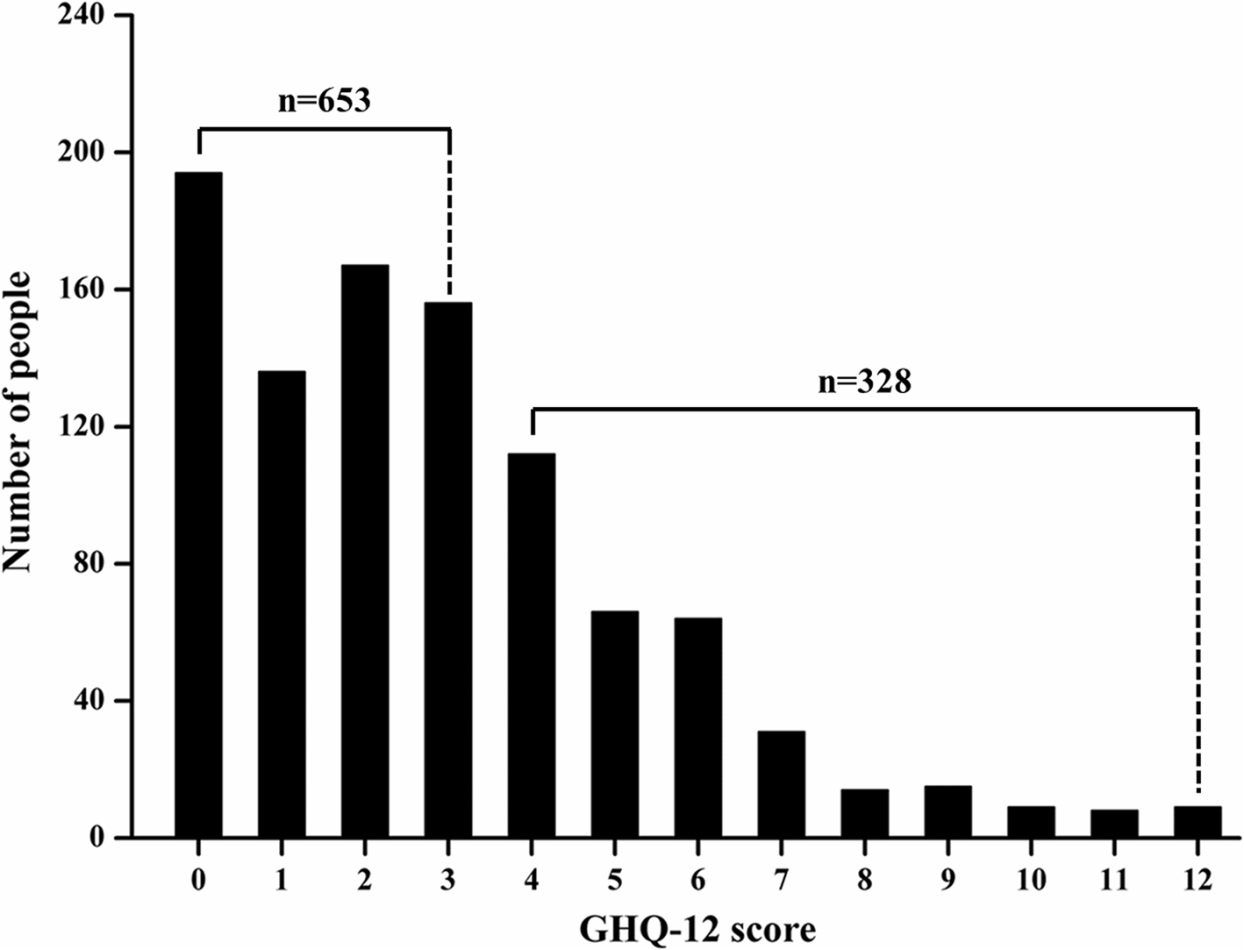
The self-reported mental health status of frontline prison officers by GHQ-12 score distribution

Previous studies have demonstrated that GHQ-12 total scores of four or above indicated with mental health problems in mainland China [29, 30]. To investigate the prevalence of psychological problems, the self-reported survey was conducted usingGHQ-12 scale. The result found that33.43% of frontline prison officers (328/981) may have mental health problems during the prevention and control of the COVID-19 epidemic. The distribution of the GHQ-12 score was shown in Fig. 1, which was not normally distributed by Kolmogorov-Smirnov test (*p*-value< 0.05).

### Psychological factors among frontline prison officers

As shown in Table 2, the mean scores were ranked higher with Factor I than with Factor II and III. Meanwhile, the highest total scores were item 9, 2, and 5. These results indicated that the frontline prison officers suffered from psychological problems associated with anxiety and depression, which main symptoms were unhappy and depressed, lost sleep over worry and constantly under strain.

**Table 2:** Psychological factors of the 12-item General Health Questionnaire

Psychological factors	Items	Total scores	Mean score of each item	Mean score of each factor
Factor I: anxiety and depression	2. Lost sleep over worry	453	0.46	352.5
	5. Felt constantly under strain	414	0.42	
	6. Felt couldn’t overcome difficulties	56	0.06	
	9. Been feeling unhappy and depressed	487	0.50	
Factor II: social dysfunction	1. Able to concentrate	188	0.19	173.5
	3. Felt playing useful part in things	115	0.12	
	4. Felt capable of making decisions	103	0.10	
	7. Able to enjoy day-to-day activities	293	0.30	
	8. Been able to face problems	35	0.04	
	12. Been feeling reasonably happy	307	0.31	
Factor III: loss of confidence	10. Been losing confidence in self	264	0.27	199.5
	11. Been thinking of self as worthless	135	0.14	

### Related factors of the mental health

Multivariable logistic regression was performed to further analyze the related factors for mental health status. As illustrated in Table 3, results of the multivariate analysis showed that male (OR = 1.573, 95% CI: 1.385–1.853), lockdown shift inside the prison (OR = 2.203, 95% CI: 2.139–2.297), more night shifts (OR = 2.163, 95% CI: 2.031–2.317; OR = 2.749, 95% CI: 2.194–2.901), more smoking (OR = 1.100, 95% CI: 1.037–2.168), poor self-reported physical condition (OR = 1.947, 95% CI: 1.478–2.250), chronic or serious illness history (OR = 1.870, 95% CI: 1.314–2.660; OR = 2.214, 95% CI: 1.460–2.812) were risk factors for mental health among frontline prison officers, while regular diet (OR = 0.779, 95% CI: 0.539–0.928), more physical exercise (OR = 0.702, 95% CI: 0.548–0.899; OR = 0.641, 95% CI: 0.316–0.887), more communication with family members (OR = 0.437, 95% CI: 0.295–0.616) were protective factors.

**Table 3:** Related factors for mental health through multivariable binary logistic regression model

Variables	Factors	*β*	OR (95% CI)	*p*-value
Gender	Female	Reference	1.000	–
	Male	0.557	1.573 (1.385–1.853)	**0.006
Working years	< 5	Reference	1.00	–
	5–10	–	–	> 0.05
	> 10	–	–	> 0.05
Recent working mode	Home quarantine and preparation	Reference	1.000	–
	Centralized isolation and preparation	–	–	> 0.05
	Work in closed jail	1.592	2.203 (2.139–2.297)	** < 0.001
Number of night shifts (times/month)	< 7	Reference	1.000	–
	7–15	1.193	2.163 (2.031–2.317)	*0.026
	> 15	1.225	2.749 (2.194–2.901)	** < 0.001
Diet	Irregular	Reference	1.000	–
	Regular	−0.405	0.779 (0.539–0.928)	*0.031
Physical exercise (days/week)	No exercise	Reference	1.000	–
	≤ 2	−0.354	0.702 (0.548–0.899)	**0.005
	≥ 3	−0.626	0.641 (0.316–0.887)	** < 0.001
Smoking	Never	Reference	1.000	–
	Sometimes	–	–	> 0.05
	Everyday	0.550	1.100 (1.037–2.168)	*0.027
Physical condition	Good	Reference	1.000	–
	Poor	0.801	1.947 (1.478–2.250)	** < 0.001
Disease history in the past year	No illness	Reference	1.000	–
	Chronic	0.626	1.870 (1.314–2.660)	**0.001
	Serious Illness	1.217	2.214 (1.460–2.812)	** < 0.001
Communication with family members (times/week)	0–2	Reference	1.000	–
	≥ 3	−0.354	0.437 (0.295–0.616)	** < 0.001

*β* Standard partial regression coefficient, *OR* Odd ratio, *95% CI* 95% confidence interval, **significant at *p* < 0.01, *significant at *p* < 0.05

## Discussion

Increasing attention has been paid on the mental health issues of frontline healthcare workers during the global public health emergency [18, 31]. Chinese prison system used control methods including creating an isolation area, suspending prison visits activities, wearing protective gear, providing training and giving public service support, there were also other distinctly unique China’s governing strategies of “lockdown shifts” to contain the spread of COVID-19 inside its walls. In general, a lockdown shift means prison officers were divided into three shift groups, with each going through three stages in loops: quarantine at designated location, lockdown shift inside the prison and a lockdown resting period at home. All Chinese prisons were put under lockdown mode, to a greater or lesser degree, from the end of January to September 2020. A latest study have demonstrated showed that prison officers were ordered to fight at the forefront of pandemic control in prisons by working on in shifts inside for an extended and indefinite period of time, proved effective in terminating the spread of COVID-19, but placed a heavy burden on the personal lives of the officers [9]. The security personnel especially prison officers who were also actively engaged in preventing and controlling this epidemic were largely neglected. To the best of our knowledge, this study was the firstly conducted to evaluate mental health status of frontline prison officers in China during the COVID-19 epidemic, and to identify factors that influence psychological distress mental health.

From March to April 2020, the entire Chinese prison system was generally under high pressure for COVID-19 epidemic prevention and control. All Chinese frontline prison officers were ordered to work on “lockdown shifts” by China’s strategy [9]. Thus, the subjects of this study were the frontline prison officers in western China, who could represent these officers from the different location and types of facilities of Chinese prison. The age of the Chinese prison officers ranges from 18 to 65 years old (working age range in China), and the age of the respondents in current study ranges from 20 to 57 years old, which of the mean age was about 30 years. The Chinese prison officers were mainly recruited by the national civil service examination, and the admission requirements have required a college degree or above in the past decade. As a result, most of China’s frontline prison officers were highly educated. In this study, we found that 74% of respondents had a bachelor degree and above. In China, there are more male prison officers than female officers in prisons dedicated to male prisoners, while there are more female prison officers in prisons dedicated to female prisoners. There are significantly more male prisons than female prisons in China, because that the female prisoners were only 8.6% (percentage of prison population) [32]. A total of 981 frontline prison officers participated in the cross-sectional Web-based survey which was considered large enough to draw definite inferences, and the ratio of women to men was 36.44%.

The current study foundthat33.43% of frontline prison officers who prevented the COVID-19epidemic from spreading to prison may have mental health problems. The finding obtained in the study was significantly less than those obtained among frontline healthcare professionals (42.67%) [6]. There was no any data and previous research evaluating the mental health of prison officers, resulting in an impossible comparison with the prevalence findings during other periods. However, respondents with prison officers during the prevention and control of the COVID-19 epidemic reported significantly higher levels of psychological problems than those with Chinese adults surveyed in 2019 (3.6–5.0%) [20]. These results reflected that frontline prison officers reached a level of mental health problems, which could not be ignored and required further evaluation.

The 12-item General Health Questionnaire (GHQ-12) was an effective instrument for screening mental disorders, and has been widely used in Chinese community samples [28, 29, 33]. Psychological factors analysis showed that frontline prison officers mainly experienced mental health issues associated with anxiety and depression, and issues related to these symptoms were unhappy and depressed, lost sleep over worry and constantly under strain. These findings were consistent with several researches, which suggested that anxiety, depression, sleep issues [34], and stress [35] were the most common psychological problems caused by COVID-19 epidemic. Excessive anxiety might lead to several harmful consequences including lower quality of life [36], physical chronic conditions [37], and relationship complications [38]. Many emotional and physical health problems, as well as decrease of the ability to function at work and home, could be elicited by depression [39, 40]. It implied that psychological intervention should be carried out for frontline prison officers early to help alleviate their psychological symptoms.

Despite it was necessary to evaluate the prevalence of psychological symptoms in frontline prison officers, the further analysis for identifying risk and protective factors contributing to mental health issues was more important. Data from the present study showed that male frontline prison officers were more likely to suffer from mental health problems compared with female prison officers. This contrasted with findings in the general population [41], healthcare workers [42], and police personnel [16] duringtheCOVID-19epidemic.Reason for the finding was attributed to men might take more responsibility leading to psychological distress symptoms [43]. The participants with lockdown shift inside the prison recently were about2.203 times (OR = 2.203) more likely to have mental health problems than that with lockdown resting period at home, which because a person in a closed environment might develop psychological problems [44]. Our results suggested that more night shifts have increased the risk of mental health problems in frontline prison officers. Consistent with earlier study, more night shifts could cause sleep disturbances, and burnout and mental disorder among employees [45]. The frontline prison officers with mental health problems smoked significantly more and were therefore at greater risk [46]. Half of this sample smoked regularly or sometimes, which was shocking. Thus, the governing strategy of Chines prison should consider including a recommendation for support for smoking cessation to be included in workplace activities, with the ultimate goal of establishing prisons as a smoke-free workplace. Moreover, participants who were in poor self-reported physical condition or had a chronic or serious illness history had a higher risk of psychological issues. It might indicate that mental health defense of this prison officer subgroup with weak physical function has been negatively affected.

Previous studies have confirmed that healthy diets and exercise regularly could promote mental health for the adult population [47, 48]. Our data proved that regular diet and more physical exercise were protective factors for mental health among frontline prison officers, and the incidence of psychological problems were 0.779 and 0.702 times that of the opposite. The present study also revealed that communication with family members was another protective factor associated with mental health of the prison officers during the prevention and control of the COVID-19 epidemic. This might be due to the fact that providing emotional support from family and friends and maintaining contact were positive treatments to protect mental health [49].

Since the early phase of COVID-19 epidemic control, the prison authorities in China have been constantly implemented psychological interventions, including publishing the mental health handbook for prison officers, establishing psychological assistance hotlines, and assigned mental health professionals to provide timely psychiatric help. These measures have efficiently alleviated the psychological problems of frontline prison officers, thereby reducing the rate of work errors. As the normalization of epidemic prevention and control will continue for a long time, this study may provide practical guidance for the development of a psychological support strategy. According to the current findings, the targeted interventions should be performed to relieve mental health problems among frontline prison officers. First, prison authorities should formulate and implement a viable strategy to adjust duty hours to the optimization, and provide solid logistical support to guarantee prison officers’ quality of life with sleep, diet and exercise, so that their physical and mental health problems can be reduced. Second, the mental health of frontline prison officers should be dynamically monitored to provide appropriate information on treatment and psychological intervention for affected people, and supply continuity of regular mental health care services among the whole population. Third, the prison officer should maintain good living habits (such as quit or smoke less, regular diet, more physical exercise) to promote mental health, and learn to use emotion regulation strategies for reducing psychological stress. Fourth, the authorities need to emphasize the role of family support during this epidemic, increase opportunities for frontline prison officers to communicate with family members, so as to avoid their psychological disorders caused by closed information between each other. Fifth, our government and relevant authorities should strengthen the psychological evaluation, online psychological counseling, psychological crisis prevention and intervention of frontline prison officers during epidemic control and in the future.

There have been several limitations in present study, which were as follows: (1) our respondents were all from China, which might not fully represent the mental health status of frontline prison officers around the world. (2) Given the particularity and complexity of COVID-19 epidemic prevention and control, we have conducted an online survey using snowball sampling approach, which the participants were limited to using smartphones or computers with network link service. (3) The mental health problems of frontline prison officers might have been biased due to recall and selection bias in self-reports on the individual’s physical and mental health. (4) This study have employed cross-sectional design, so only association analyses allowed and causal inferences could not be made, and it only revealed the psychological status of frontline prison officers during a certain period of epidemic control. (5) The factor analysis used in this study had a serious limitation: there was no objective and uniform standard for determining the number of factors. Meanwhile, the lack of control group in the current study made it impossible to analyze the comparison of mental health problems between frontline and non-frontline prison officers during the prevention and control of the COVID-19 epidemic. Future research should adopt longitudinal design to evaluate the long-term psychological implications of the epidemic through exploring multiple time points.

## Conclusion

To sum up, the present study indicated that Chinese frontline prison officers experienced different psychological pressures coming from the prevention and control of the COVID-19 epidemic. This study also suggested that being male, lockdown shift inside the prison, night shifts, smoking, self-reported physical condition, history of chronic or serious illness, regular diet, physical exercise, communication with family members were the influence factors of associated with mental health. Previous studies confirmed the importance of occupational health surveillance to monitor mental health conditions of workers during and after COVID-19 pandemic [50, 51]. The findings also highlighted that the need for prison authorities to continuously monitor the psychological impact of public health emergency emergencies on frontline prison officers, and timely develop and implement timely targeted interventions to tackle mental health problems.

## Supplementary Information

**Additional file 1.** Survey questionnaire on mental health status of frontline prison officers during the prevention and control of the COVID-19 epidemic.

## Data Availability

The study data used and analyzed in this study are available from the corresponding author on reasonable request.

## Abbreviations

COVID-19: Coronavirus Disease 2019
GHQ-12: The 12-item General Health Questionnaire
OR: Odds ratios
95% CI: 95% confidence interval
