# Supplementary material for: Mental health status among prison officers in the process of enforcing the law during COVID-19epidemic: a cross-sectional survey from China
Source: BMC Psychiatry. 2022 Jan 11;22:33. doi: 10.1186/s12888-021-03679-0 (PMC8749117; doi:10.1186/s12888-021-03679-0)
Supplement: Supplementary file 1 — Additional file 1. Survey questionnaire on mental health status of frontline prison officers during the prevention and control of the COVID-19 epidemic. [file 12888_2021_3679_MOESM1_ESM.docx]

**Additional file 1** Survey questionnaire on mental health status of frontline prison police during the prevention and control of the COVID-19 epidemic.

| Parts | Question | Answers |
| --- | --- | --- |
| Part I: informed consent form | 1. The survey was anonymous, and the data obtained would only be used for subject research. The right to participate voluntarily was absolute. Do you agree to participate in the questionnaire survey? | Yes |
|  |  | No |
| Part II: socio-demographic section | 2. What is your gender? | Female |
|  |  | Male |
|  | 3. How old are you? |  |
|  | 4. Are you married? | Yes |
|  |  | No |
|  | 5. What is your highest educational background? | College degree and below |
|  |  | Bachelor degree and above |
|  | 6. How long have you worked in prison? | < 5 |
|  |  | 5-10 |
|  |  | > 10 |
| Part III: work and life situations | 7. What is your recent work model? | Home quarantine and preparation |
|  |  | Centralized isolation and preparation |
|  |  | Closed on duty |
|  | 8. How many night shifts do you work in a week? | < 7 |
|  |  | 7-15 |
|  |  | > 15 |
|  | 9. How about your diet recently? | irregular |
|  |  | regular |
|  | 10. How many times a week do you physical exercise? | No exercise |
|  |  | ≤ 2 |
|  |  | ≥ 3 |
|  | 11. How often do you smoke? | Never |
|  |  | Sometimes |
|  |  | Everyday |
|  | 12. How is your physical condition? | good |
|  |  | poor |
|  | 13. What is your disease history in the past year? | No or minor illness |
|  |  | chronic |
|  | 14. How many times did you communicate with family members in a week? | 0-2 |
|  |  | ≥ 3 |
| Part IV: 12-item General Health  Questionnaire (GHQ-12) | 15. Have you recently been able to concentrate on what you are doing? | Better than usual |
|  |  | Same as usual |
|  |  | Less than usual |
|  |  | Much less than usual |
|  | 16. Have you recently lost much sleep over worry? | Not at all |
|  |  | No more than usual |
|  |  | Rather more than usual |
|  |  | Much more than usual |
|  | 17. Have you recently felt you were playing a useful part in things? | Better than usual |
|  |  | Same as usual |
|  |  | Less than usual |
|  |  | Much less than usual |
|  | 18. Have you recently felt capable of making decisions about things? | Better than usual |
|  |  | Same as usual |
|  |  | Less than usual |
|  |  | Much less than usual |
|  | 19. Have you recently felt constantly under strain? | Not at all |
|  |  | No more than usual |
|  |  | Rather more than usual |
|  |  | Much more than usual |
|  | 20. Have you recently felt you couldn’t overcome your difficulties? | Not at all |
|  |  | No more than usual |
|  |  | Rather more than usual |
|  |  | Much more than usual |
|  | 21. Have you recently been able to enjoy your normal day-to-day activities? | Better than usual |
|  |  | Same as usual |
|  |  | Less than usual |
|  |  | Much less than usual |
|  | 22. Have you recently been able to face up to your problems? | Better than usual |
|  |  | Same as usual |
|  |  | Less than usual |
|  |  | Much less than usual |
|  | 23. Have you recently been feeling unhappy and depressed? | Not at all |
|  |  | No more than usual |
|  |  | Rather more than usual |
|  |  | Much more than usual |
|  | 24. Have you recently been losing confidence in yourself? | Not at all |
|  |  | No more than usual |
|  |  | Rather more than usual |
|  |  | Much more than usual |
|  | 25. Have you recently been thinking of yourself as a worthless person? | Not at all |
|  |  | No more than usual |
|  |  | Rather more than usual |
|  |  | Much more than usual |
|  | 26. Have you recently been feeling reasonably happy, all things considered? | Better than usual |
|  |  | Same as usual |
|  |  | Less than usual |
|  |  | Much less than usual |
